# Relationship between Clinical Symptoms and Magnetic Resonance Imaging in Temporomandibular Disorder (TMD) Patients Utilizing the Piper MRI Diagnostic System

**DOI:** 10.3390/jcm10204698

**Published:** 2021-10-13

**Authors:** Tor Tegnander, Grzegorz Chladek, Anders Hovland, Jarosław Żmudzki, Piotr Wojtek

**Affiliations:** 1 Tor Tegnander Dental Clinic, Tollbugata 10, 8006 Bodø, Norway; Tor.Tegnander@tanntor.no; 2 Department of Engineering Materials and Biomaterials, Faculty of Mechanical Engineering, Silesian University of Technology, Konarskiego 18A Street, 44-100 Gliwice, Poland; jaroslaw.zmudzki@polsl.pl; 3 Division of Internal Medicine, Nordland Hospital, Parkveien 95, 8092 Bodø, Norway; anders.w.hovland@gmail.com; 4 Polish-Norwegian Medical Consultants, ul. Mickiewicza 5, 44-100 Gliwice, Poland; pmwojtek@gmail.com

**Keywords:** temporomandibular joint, temporomandibular disorders, symptoms, pain, Piper MRI diagnostic system

## Abstract

Clinical problems of the temporomandibular joint (TMJ) and the masticatory musculature are both included in the term temporomandibular disorder (TMD). The purpose of the present study was to examine the pathology of the joints of patients diagnosed with TMD utilizing the dedicated Piper MRI-based classification, and to link these pathologies with various symptoms while considering their severity. In total, 64 patients with clinical TMD were examined. Symptoms were recorded using a questionnaire. The clinical examination included diagnosing the occlusion in centric relation, which was followed by a standardized MRI. It was confirmed that, although they occurred in a high percentage in all classes, muscle pain and occlusal interference are not indicators of TMJ damage. The results indicate that the progressive degradation of the TMJ, represented by qualification to the higher Piper classes, is associated with an increase in TMJ pain only up to a certain stage. For the highest Piper classes, the joint pain occurs in a smaller percentage of patients, but sounds are more frequent.

## 1. Introduction

The typical reasons for visits to the dentist are dental caries, periodontal problems, or, more rarely, the loss of teeth [1]. Clinical problems related to the temporomandibular joint (TMJ) and the masticatory musculature, both included in the term temporomandibular disorder (TMD), are not typically reported by patients. TMD is not qualified as life-threatening. However, it may influence quality of life [2] due to the fact that symptoms may become chronic if not taken care of. Moreover, with time, TMD symptoms may become difficult to treat. The etiology is recognized as multifactorial, but the most common factors are considered to be predisposing factors (systemic, structural, psychologic, or genetic factors, which increase the risk of TMD/orofacial pain), initiating factors (e.g., trauma or an overloading joint structure, which causes the disorder to begin), and perpetuating factors (e.g., mechanical, muscular stress, or metabolic problems, which interfere with healing or complicate treatment) [3,4,5]. All of these factors may influence each other and/or act together [3,5], which creates an additional challenge for successful treatment.

TMD is considered a collective term for different symptoms, including pain in the TMJ, headache, teeth clenching, grinding, impaired mouth opening, clicking, crepitus in the TMJ, and masticatory tenderness [6]. Patients with TMD symptoms are diagnosed over a broad age range, but a peak occurs between 20 and 40 years of age [7]. The prevalence of TMD is greater than 5% of the population, and some research suggests that between 6% and 12% of the population experiences the clinical symptoms of TMD [8,9,10]. Several symptoms, including TMJ crepitation, facial/jaw pain, headache, teeth clenching, grinding, impaired mouth opening, clicking, crepitus, TMJ tenderness, and masticatory muscle tenderness, have been described [11]. However, previous studies have concluded that structural changes may occur in the TMJ of persons with no symptoms of TMD [12]. The treatment of the broadly understood ailments associated with TMD remains difficult because different symptoms are not necessarily connected. Therefore, there are many available, often varied and multi-level, methods used to help patients, but their effectiveness varies and is uncertain [13,14,15]. Moreover, specialists recognize this problem. This is evidenced by the fact that, in some regions, only 6.5% of the dentists identified their TMD knowledge as very good [16], and 95.4% of them said they were interested in attending a TMD continuous education program [17]. Because of this, the precise treatment is not always administered to patients with TMD, indicating a need for further research in the field [18].

TMD is considered to have multiple etiological factors. Although the research diagnostic criteria for temporomandibular disorder (RDC/TMD) has been established [19], patients with similar signs may show a normal physical variation, a preclinical state, or a disease state. Even in the case of support through vibrational diagnostics, it is not possible to unequivocally diagnose 15–25% of cases [20,21]. The occurrence of pain symptoms, along with the severity of degenerative joint disease, is particularly controversial [22,23,24,25,26,27,28,29,30,31,32]. Significant relationships were found between TMJ crepitation and mean osseous changes (diagnosed and calculated in four radiographic characteristics based on the RDC/TMD) in osteoarthrosis (without pain) and osteoarthritis (with pain) groups [30]. However TMJ osseous changes do not correlate significantly with TMJ pain [30,31,32,33]. RDC/TMD based on radiographic characteristics of osseous changes can be aided with the visualization of changes in the disks using MRI [34]. Piper introduced a system for analyzing the MRI scans of the TMJ and a classification system for the diagnosis of disk displacements, which is a further development of the Wilkes system, with both describing five stages of destruction of the TMJ [20,35]. It is suggested that RDC/TMD system is especially useful for specialists in the orofacial pain practice. However, for a restorative dentist or interdisciplinary specialist, the Piper system may be recommended [36,37]. There have been numerous studies on the MRI findings of the TMJ [12,38], but the classifications are still insufficiently related to the clinical symptoms. Disk changes can be seen in up to 80% of TMD patients, but 30% of asymptomatic cases have similar findings [12,39,40,41].

The purpose of the present study was to examine the pathology of the joints of patients diagnosed with TMD utilizing the dedicated Piper MRI-based classification, and to link these pathologies with various symptoms while considering their severity.

## 2. Experimental Section

### 2.1. Inclusion/Exclusion Criteria and Clinical Protocol

In the study, 64 patients were included, and all patients provided their informed consent. All patients came for treatment in the private dental clinic Tollbugata in Bodø, Norway.

The clinical examinations were conducted and considered the inclusion criteria of joint disorders [19,20,42]. The inclusion criteria were as follows:

- Painful TMD, with a diagnosis based on the previously published criteria for TMD [19,20,42]
- Facial pain noted at least 3 months prior to the visit
- Full dental arches (previous normal dental treatment without implants)
- Orthodontic treatment was ended at least one year ago
- General good health
- At least 18 years of age
- Fluent in Norwegian or English.
- The exclusion criteria were as follows:
- Undergoing or completed orthodontic treatment in the last 12 months
- Previous craniofacial surgery and/or noted injuries
- The patient was not willing to undergo MRI or not suited for MRI
- Cognitive impairment
- Psychiatric limitations that may affect the participation in the study assessments

The final patient group consisted of 15 men, 20–77 years old, and 49 women, 20–66 years old. The mean age of the total group was 38.9 years. In total, 128 joints were examined, of which 68 joints were classified as IVa class, 22 as IVb, 12 as Va, and 22 as Vb. In addition, for one patient, the joints were classified as class II and IIIa. Because it was a single case, it was not included in the presented study.

The questionnaire/evaluation of symptoms was completed in the clinic with the examining dentist assisting the patient (the patients had previously received the questionnaire so they could be acquainted with the questions). During the visit, clinical interview was performed, and the symptoms were described. It was determined whether patients suffered pain (continuous or intermittent) from headaches, or their neck, jaw, ear, face, or another region. The painful regions were marked on a diagram showing the right and left profile schematically. It was determined how long the patient experienced the pain, whether it was constant or intermittent (aching burning, stabbing, or other), and whether it occurred in the morning, afternoon, evening, or/and at night. It was determined whether the patient had sustained any injuries in the past, what made the pain better or worse, and whether the patient was using medications to improve the situation (medication, dose, and frequency). It was established whether and in what situations the patient experienced pain (while chewing, and if he/she experienced popping/clicking/other noises on the right or left side). It was determined whether the patient’s jaw ever locked, and whether the patient noticed changes in the occlusion (front or back teeth), the profile, or the asymmetries in the maxilla/mandible. It was determined whether the patient had excessive tooth sensitivity, or had ear problems such as reduced hearing, ringing, dizziness, or other problems. It was determined if the patient had swallowing problems; if/when he/she had any TMJ-related treatment such as a splint, night guard, occlusal adjustment, or orthodontic treatment; and whether these treatments were effective. The muscles were palpated to determine which were painful. The dentist palpated the jaw muscles bilaterally to discover sore trigger points and sense the muscle tonus. Masseters were palpated with the fingers positioned over the angle of the mandible, the temporalis muscles were palpated along the temple with the jaw relaxed and clenched, and the pterygoid muscles were palpated intraorally along the medial aspect of the mandibular ramus between the tonsillar pillars [8]. The sub-occipital muscles and the musculus trapezius were also palpated. The latter is particularly important because it may, through the sensory cortex, give rise to heterotropic (referred) pain in the TMJ area.

The protocol followed the criteria of The Helsinki Declaration, ICH Guideline for Good Clinical Practice. The study was approved by the ethical committee of the University of Bialystok (R-1-002/1, 2/2016).

The occlusal interference was diagnosed in centric relation (CR), defined as the most anterior superior position of the condyle disk assembly within the glenoid fossa. The CR was recorded by bimanual manipulation. The models were prepared, placed in this position, and mounted in an articulator (Artex Amann Girrbach CN, Amann Girrbach, Koblach, Austria). The occlusal interference was recorded on the model and on the patient utilizing articulating paper (PD Dentaire Switzerland, thickness of 50 µm).

### 2.2. MRI Imaging and TMJ Classification

The MR imaging was obtained with a GE Signa 1.5 T MR scanner (General Electric, Chicago, IL, USA), with a dedicated coil for a TMJ protocol. All MRI scans were read by the dentist and two experienced radiologists. The radiologists were blinded to the clinical data. The differences in the assessment carried out by specialists concerned only the details and did not affect the final diagnosis/qualification, which was the same in all cases. The following factors were determined as normal or abnormal: condylar translation (restricted was abnormal), condyle cortex (sharp was normal, poorly defined or sclerotic were abnormal), condyle shape (round was normal, flat and/or irregular were abnormal), condyle size (osteochondrosis—small, hyperplasia—enlarged, or AVN—regressed were abnormal), articular spacing (increased or decreased were abnormal), disk quality (enlarged, mild degeneration, moderate degeneration, or severe degeneration were abnormal), disk posture (Figure, 1 o’clock was normal, 12 o’clock, 10 o’clock or 11 o’clock were abnormal), and occlusal interference.

The TMJ MRI scan protocol was as follows: the sagittal slices should cut perpendicular to the line bisecting the medial and lateral poles; the coronal slices should be parallel to the line bisecting the medial and lateral poles; T1 (TR 500–700, TE 15–30); sagittal slices were performed with closed-mouth CR, and when possible, with an open mouth (30 mm minimum); the coronal slices were obtained with a closed mouth; T2 (TR 2200–3000; TE 80–100); the sagittal slices were performed utilizing the proton density (TR 2200–3000, TE 15–30).

**Figure 1:**
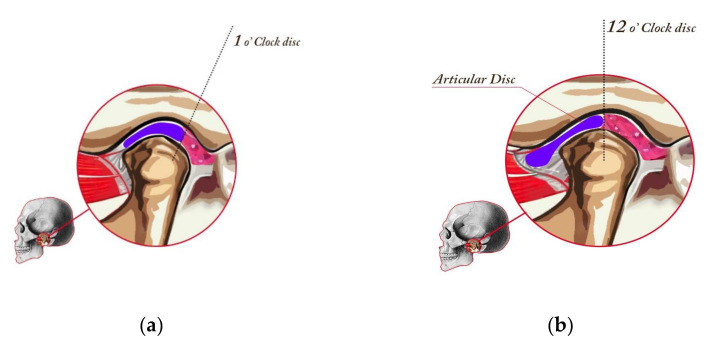
Healthy joint (stage I in the Piper classification), placed in CR, and drawing of a cut at the medial pole. Posterior band of the disk aligned with the schematic 1 o’ clock position (**a**) and minor change in the disk position, which constitutes the Piper IVa classification. The joint is in CR, and the drawing at the medial pole of the TMJ. In class IV, the disk slips in the anterior direction when opening the jaw (**b**).

The examined TMJ stages were divided into five groups according to the Piper classification [35], which was constructed on the basis of the MRI findings, including the following criteria:

Stage I—Normal (Figure 1a). In a normal temporomandibular joint, there are tight collateral ligaments. These ligaments bind the disk to the lateral and medial poles, respectively. The collateral ligaments are functionally and structurally intact. The disk is postured so that the posterior band is just proximal (located toward the ear) to the mid part of the fossae (“1 o’clock position”).

Stage II—Intermittent Click. The earliest soft tissue breakdown within the temporomandibular joint occurs at the lateral pole. Note that the disk is in normal alignment most of the time. Approximately 25 percent of the temporomandibular joints have lateral pole laxity, or Piper Stage II. This is by far the most common internal arrangement.

Stage IIIa—Lateral Pole Click. There is a more chronic displacement of the disk from the lateral pole. Fibrosis begins to develop in the lateral part of the superior belly of the lateral pterygoid muscle. This results in chronic clicking because of the displacement of the disk from the lateral pole of the condylar head.

Stage IIIb—Lateral Pole Lock. Stage IIIb is the locking phase of lateral pole disk disease. In this stage, the lateral 50% of the disk is dislocated from the condylar head. Therefore, there is no joint clicking. It is probable that as much as 15% of the global population has Stage IIIa or IIIb disk displacement. Because the medial pole is covered by the disk, these joints tend to be more comfortable when they are returned to centric posture (4).

Stage IVa—Medial Pole Click (Figure 1b). Stage IVa disease involves the displacement of the disk from the medial pole of the condylar head. The clicking phase of the medial pole disk displacement is stage IVa. Most of these patients have simultaneous displacement of the disk from the lateral pole. Stage IVa joints are more likely to develop intra-articular pain because both translatory and rotary movement will load retrodiscal attachment.

Stage IVb—Medial pole lock. Stage IVb is the locking phase in patients who have medial disk displacement. There has been significant deformation of the disk at the medial pole.

Stage Va—Perforation with acute disk joint destruction (DJD).

Stage Vb—Perforation with chronic DJD. These patients have chronic disk displacement and perforation of the retrodiscal tissues.

### 2.3. Statistical Analysis

Statistical analysis of the results was performed using the PQStat 1.8.0 software (PQstat Softwere, Poznań, Poland). The Pearson’s chi-square (χ2) test for categorical data or Fisher’s exact test (α = 0.05) was used. The sample size was calculated. The effect size (Cramér’s phi, φc) was assumed as medium (φc = 0.3) or large (φc = 0.5) [43]. For 4 × 2 tables (power 0.8, effect size 0.3–medium effect, α = 0.05), the calculated sample size was 122 joints, and for 4 × 4 tables (power 0.8, effect size 0.5–large effect, α = 0.05), it was 44 joints. Ultimately, it was possible to analyze a larger number of joints (*n* = 128). Considering that the obtained results for each of the patients were considered valid, we included them in the analyses. As a result, for tables 4 × 4 (effect size 0.5–large effect, α = 0.05), the calculated power was 0.99, and for tables 4 × 2 (effect size 0.3–large effect, α = 0.05), the calculated power was 0.82.

## 3. Results

A total of 10 patients (16%) had additional medical disorders, and some patients had several disorders. The most prevalent disorder was medically treated hypertension in five patients (8%), while three patients had asthma and two had rheumatism. Individual cases of diabetes, cancer treatment, or occasional depression an intermittent dose antidepressant) were registered.

In total, 24 patients suffered from headaches (38%), 28 felt neck pain (44%), 5 had problems with locking joints (8%), and 4 suffered from ear fullness (6%). It was also found that 52% of patients (33 of 63) could link TMD to previous trauma. MRI scans disclosed that all patients had changes in their TMJ, and all patients also had pathological changes corresponding to various degrees of disk displacement. All patients suffered from painful jaw muscles (left or right side or both sides). It was found that 58 patients (92%) of the patients complained of pain in the masseter muscle, 36 (57%) had temporal muscle pain, and 35 (55%) had other jaw muscle pain. Joint pain was noted for 14 (22%) patients, and 36 (57%) had joint sounds.

### 3.1. The Correlation of Piper Classification on the Symptoms Related to TMJ Functioning

**Figure 2:**
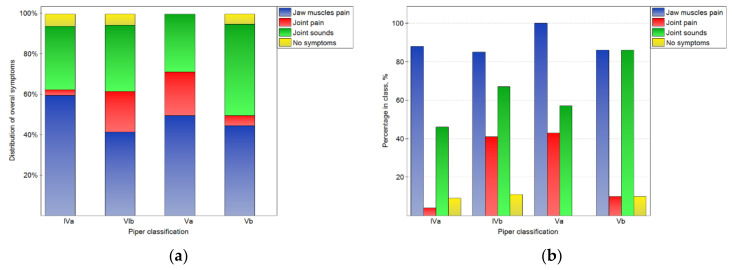
Impact of the Piper classification on the chosen overall symptoms related to TMJ functioning: the distribution of symptoms among the classes (**a**), and the percentage of symptoms in the number of noted cases for the particular classes (**b**).

**Figure 3:**
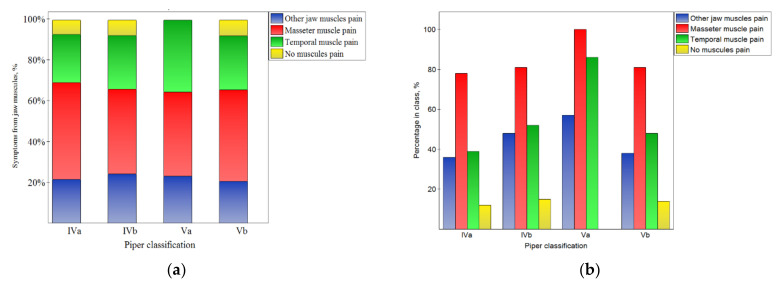
Impact of the Piper classification on the symptoms from muscles: the distribution of symptoms among the classes (**a**), and the percentage of symptoms in the number of noted cases for the particular classes (**b**).

**Figure 4:**
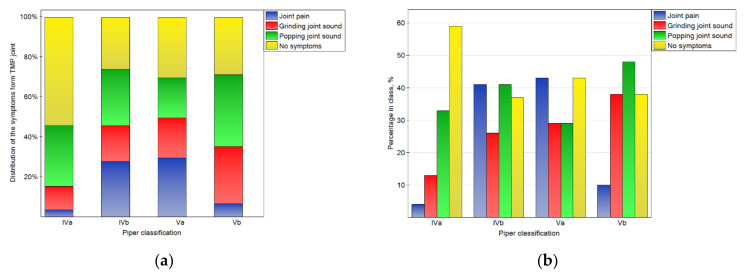
Impact of the Piper classification on the symptoms from TMJ: the distribution of symptoms among the classes (**a**), and the percentage of symptoms in the number of noted cases for the particular classes (**b**).

The analysis of the results depends on the side that the joint was located (shown in Figure 2, Figure 3 and Figure 4). In Figure 2a, the distributions of overall symptoms from the muscles and the TMJ depending on the Piper classification are presented. Statistically significant (*p* < 0.05) differences in the distribution of the symptoms were noted. These results may be analyzed together with the percentage of symptoms in the number of cases for the particular Piper classes (2b). For classes Iva–Va, muscular pain was the most frequently noted symptom, and the pain was noted for more than 85% of the joints in all Piper classes. However, in class IVb and Va TMD, joint pain was reported frequently, but for classes IVb and IVa, it was registered from four- to ten-times less often, respectively. At the same time, for class Vb, sounds from the joints were noted in 86% of cases. However, sounds from the joints were noted much less frequently in the other classes. Therefore, it should be noted that, for the Vb class, the most characteristic symptom was joint sounds without joint pain, but for classes IVb and Va, joint pain was the typical symptom.

This corresponds well with the results presented in Figure 3 and the fact that the pain of particular muscles was not related to the Piper classification (*p* > 0.05).

The impact of the Piper classification on temporomandibular joint symptoms is presented in Figure 4. There were statistically significant (*p* < 0.05) differences in the distribution of symptoms in the classes. For the IVa class, the symptoms were not registered in the case of most of joints. However, for the other classes, the percentages of joints without symptoms were one-third lower. Joint pain was typical for classes IVb and Va, but rare for others. Popping joint sounds were frequently (nearly half of the time) noted for the Vb class.

### 3.2. The Influence of the Piper Classification on Magnetic Resonance Imaging Results

**Figure 5:**
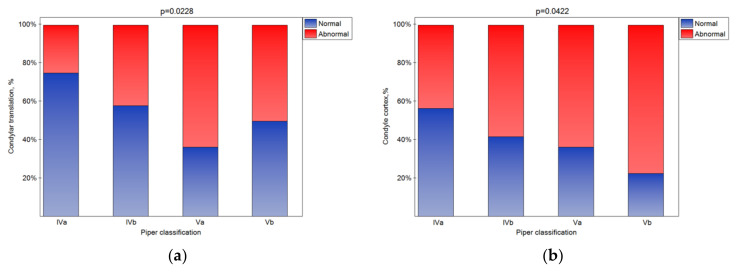

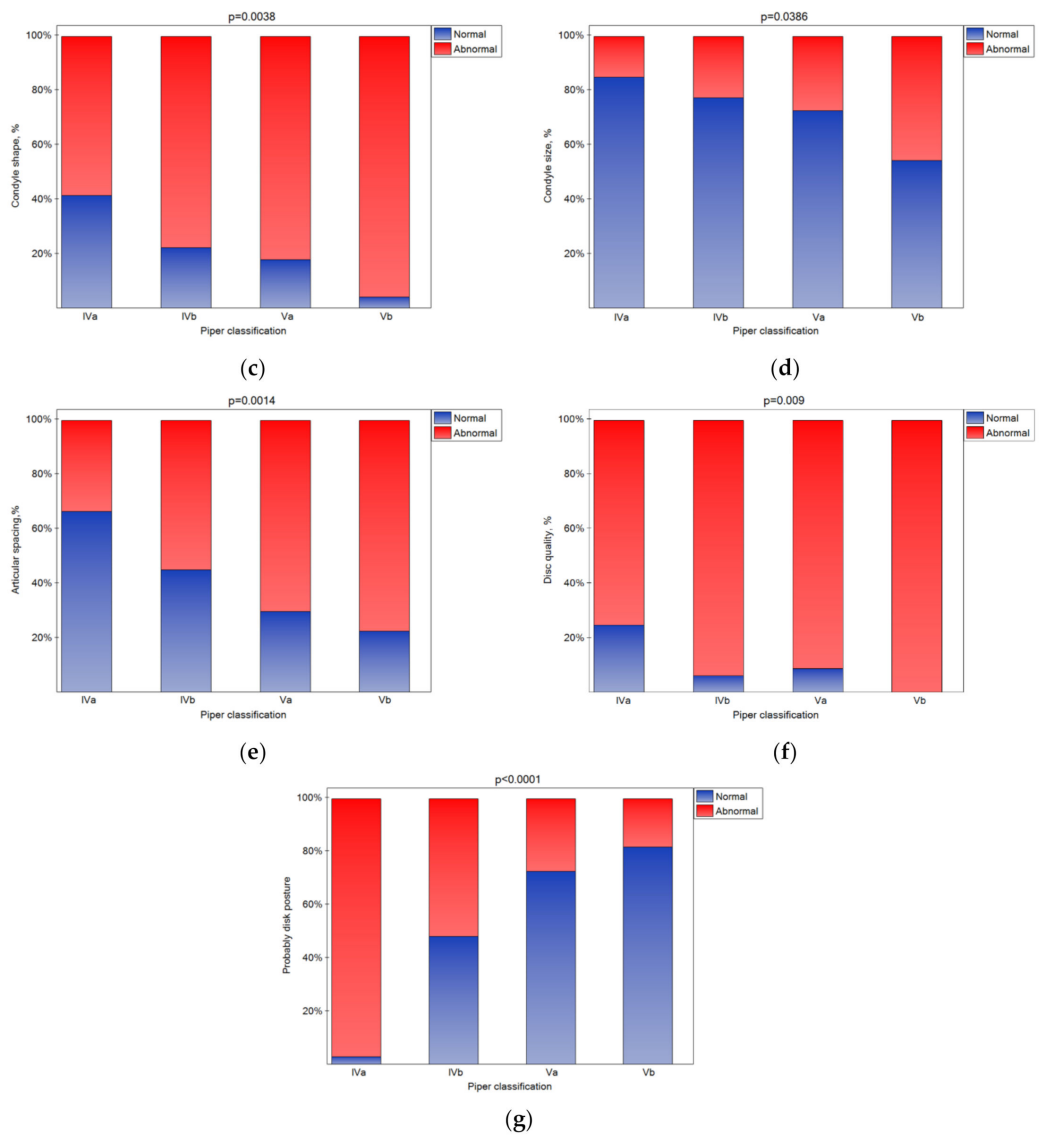
Impact of the Piper classification on the condylar translation (**a**), condyle cortex (**b**), condyle shape (**c**), condyle size (**d**), articular spacing (**e**), disk quality (**f**), and disk posture (**g**) (statistically significantly effect was for *p* < 0.05 level).

The bone changes in TMJ intensified in the subsequent classes (Figure 5a–e (statistically significant)). The condylar cortex, shape, size, translation, and articular spacing indicated bone tissue remodeling. In the Vb class, there was no normal disk quality, while in class Iva, 20% of the disks were found to have normal quality. The impact of the Piper classification on the disk quality was statistically significant (Figure 5f). As the class increased, the number of disks classified as normal significantly increased (Figure 5g (*p* < 0.05)).

### 3.3. The Influence of the Piper Classification on Occlusal Interference

**Figure 6:**
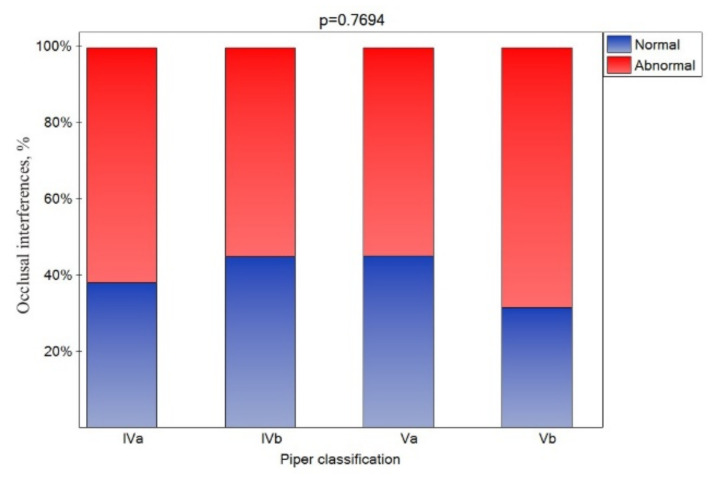
Impact of the Piper classification on the occlusion interference (statistically significant effect was assumed for *p* < 0.05).

An occlusal interference was registered in all patients. However, considering but the side of the particular joints, a CR molar occlusal interference was noted for nearly 60% of the joints (Figure 6), and its presence was similar for all Piper classes.

## 4. Discussion

### 4.1. MRI Accuracy

The disk changes and position are the key features that are diagnosed with MRI. Diagnosis depends on the technique to a large extent. An MRI at 3.0 T shows significantly better visibility and better overall image quality compared to 1.5 T [44,45] because of the spatial resolution at 3.0 T is considerably higher compared to 1.5 T [44]. It has also been reported that a diagnostic accuracy at 3.0 T is better in patients with anterior disk displacement [46]. The authors of [47,48] recommended the multisection two planes sagittal and coronal images to avoid false-negative diagnoses at 1.5 T. In a single projection, there were 15.4% more cases with normal disk position compared with oblique sagittal and coronal scans in patients with TMD. Unfortunately, the relationship between the imaging findings and the type and intensity of clinical symptoms was not included. It has been suggested that the weak correlation between clinical symptoms and imaging finding results from insufficient MRI accuracy, and can be resolved with the feasibility of imaging the TMJ at higher field strengths of 7.0 T [49]. However, so far, the 7.0 T protocol has not been verified in terms of the clinical effectiveness of TMD diagnostics on a wide group of patients. Instead, only better imaging resolution has been demonstrated, which suggests greater efficiency in detecting smaller the disks changes [50,51]. Despite the better efficacy at higher field strengths, the imaging resolution of 1.5 T is still the most available.

### 4.2. Clinical Symptoms and Imaging Findings

In the presented research, TMJs classified as Piper class IVa–Vb were analyzed due to the small number of joints assigned to classes II–III. In another study, the majority of joints were classified as classes IIIa–IIIb, but the number of joints classified as Piper II class was also small. Moreover, a much smaller number of joints were classified as Iva–Vb [20]. This fact should be noted because it means that, in the current study, only the joints with a high degree of damage were analyzed. The reasons for the differences in the distribution could vary from cultural conditions (no treatment attempts with minor symptoms) to a lack of success with prior treatments. This may be also related to the fact that a population of very young patients participated in the study [20] (the average age was 19 versus 38.9 years in our work). The fact that the analyses showed joints with a significant degree of damage may be the reason for the atypical percentage of patients experiencing pain from muscles, joint sound/pain, neck pain, and headaches [20,52,53,54]. The most common complaint was muscle pain, which was often accompanied by joint sounds, followed by joint pain. All patients had different anatomical changes in their TMJ on MRI. Some of these changes were very subtle, such as minimal anterior dislocation of the intra-articular disk. This group of patients manifested serious clinical symptoms and was classified as group IVa in the Piper classification (55% of the joints in our study). The other difference is that, in our study, relatively low figures of ear fullness were registered. This symptom occurs when the petrotympanic fissure brings the temporomandibular joint into contact with the middle ear. Inflammation can spread, beginning from the joint capsule to the origin of the levator and the tensor palatini muscles, and finally to the cul-de-sac over the isthmus of the Eustachian tube, causing the obstruction of the tube, which may be responsible for the feeling of ear fullness [55] The percentage noted in our investigations was lower than that reported by Kitsoulis et al. [56] or Kaygusuz et al. [57] (approximately 13%), as well as the percentages reported in numerous other studies, where the presence of ear fullness ranged from 20% to 90% [58]. This shows that the percentage of ear fullness can be very different and depends on many factors.

Generally, it should be assumed that a higher stage in the Piper classification would cause more frequent symptoms and more significant anatomical changes than bone tissue remodeling (condylar cortex, shape, size, translation, and articular spacing). For example, Larheim et al. found that TMJ pain patients generally had more severe joint changes compared to the asymptomatic control group [12]. Anterior disk displacements, particularly without reduction, have also been related to the presence of pain [22,23,24,25]. However, in other studies, anterior disk displacement did not necessarily correlate with joint pain [26,27,28,29]. In our work, the more severe Piper class was related to more normal disk posture. Less pain sensations were noted only in the most damaged joints (Vb class), rather than the two previous classes. However, the intensity of the problem was similar to that in class IVa. It should be noted that, despite the lower frequency of joint pain occurrence in patients classified as class Vb, the joint sounds intensified. The reference of our results to RDC/TMD indicates that the Piper classes correspond to the severity of cases classified as degenerative joint disease. The highest class includes the most cases without pain, but with more severe acoustic signals, which are classified as “osteoarthrosis” [59,60]. Our results are partially consistent with the results found by the authors of [30,31,32], in which the degree of TMJ osseous changes did not correlate significantly with clinical pain symptoms. This is partly because we did not divide the patients into painful (“osteoarthritis”) and painless (“osteoarthrosis”). Instead, the division was based on the intensification of the pathological changes in the joints. With such a division, the number of pain patients increased with the joint changes. Therefore, our results do not confirm that bone changes do not correlate with pain. On the other hand, the finding of severe TMJs changes without any clinical symptoms except joint sounds motivates research into the causes of the functioning of the system, despite the deviations from the norm which are considered anatomically correct. Some previous studies has indicated a poor correlation between the severity of TMD-related pain complaints and the evidence of definitive tissue pathology [61,62,63]. The pain described by the authors of [64] was not related to the MR findings of effusion in the internal derangement and synovial fluid aspirate findings of the total protein concentration. In addition, the authors of [65] observed no significant differences in the TMJ function in the group with systemic sclerosis compared to the group with psoriatic arthritis and the healthy controls.

The phenomenon of reducing the frequency of joint pain symptoms with the intensification of the destruction of joint structures and sounds from the joint can be explained by the progressive adaptation of the structures to the new (pathological) situation. In this state, significant damage to the adjacent structures occurs, but the situation becomes stabilized. The findings are in agreement with the results found by the authors of [66], who stated that TMD is a chronic disease that necessitates tracking of the osseous change and remodeling process over time, which can lead to an adaptive response of the joints [67]. In accordance with the Piper classification in class Vb, the joint dimensions are suspected to be stable [35]. In this pathological stage, with extreme disk destruction, the structures may collide to a lesser extent, which leads to less pain in the joint itself, but also to the intensification of sounds. It should be noted, however, that this is only a supposition that requires confirmation in further research. The absence of joint clicking and crepitation is not an indication of a normal joint, and the presence of joint sounds is not a disease indicator. Crepitation is usually related with arthrosis. However, joints with extensive remodeling (without arthrosis) can also crepitate [68] which is consistent with our findings. It is difficult to relate the intensification of sounds to the results of the joint vibration analysis. The joint vibration analysis showed that sounds with a higher frequency were observed more frequently in the pathological bone changes group (including erosion, osteophyte formation, and deformity) than in the adaptive bone changes group (including flattening and concavity) [69]. In addition, the association between bruxism and TMJ sounds [70,71] is difficult to relate to our results.

On the other hand, neuroimaging revealed that changes in the brain are associated with changes in the TMJ and MM regions and with TMD pain [72]. Muscle pain occurs at all stages in a comparable range. Moreover, patients with higher stages in the Piper classification had minor changes in disk posture. Disks that were completely dislocated and appeared anatomically healthy were also found. In the authors’ opinion, this might be explained by a trauma that knocked the disk straight off the condyle head. Other disks were progressively dislocated and completely destroyed by micro trauma, e.g., during clenching and bruxism [73]. However, these disks were originally not completely knocked off. The etiology of these problems may vary. In this context, trauma (also in childhood) [74,75,76], including the genetic predisposition for pain and/or reduced collagen repair [77,78,79] and/or fibromyalgia [80], is likely.

### 4.3. Occlusal Factors

We found occlusal interference in all patients, although these problems did not often occur on both sides—only about 60% of TMJ occlusal interference was recorded on the side of the analyzed joint without regard to the Piper class. The occlusal changes may be related to a change in the joint area because the medial part of the joint, which we examined by MRI, is the load bearing part of the joint, so occlusal changes would be expected. This can partially explain the term occlusal disease. However, it should be noted that only a couple of the selected studies have indicated a correlation between TMD and occlusal factors [81,82]. Only a few works have indicated that correctly performed occlusal adjustments may help to treat TMD [83,84], but occlusal adjustments are not usually recognized as a beneficial method in the management of TMD [14,85]. The lack of an association between TMDs and occlusion interferences indicates that these interreferences may result from TMDs [86,87,88,89]. Consequently, patients may shift their mandible as a result of the biomechanical changes of their joints [90,91] to articulate in a less painful position [92].

### 4.4. Limitations

The small size of the patient group was a limiting factor. The patients were treated in a private clinic from a geographic area with a low population density, which could have affected the results. Another limitation is that our work is retrospective, and the sample size was not determined prior to testing. The number of patients and the distribution of TMJ classified into a particular class will not necessarily be the same in other populations. In the future, a larger group of patients, including a control group of pain-free patients diagnosed according to the Piper classification, should be used. Another limitation is the fact that patients belonging to Piper classes I–III were not included. The number of people who came to the clinic was too small to include patients from classes I–III in the analyses.

## 5. Conclusions

The presented results linking these pathologies with various symptoms, considering the severity of these pathologies, add to the discussion on this subject. The results of the research indicate that the progressive degradation of the TMJ, represented by the qualification to the higher Piper classes, is associated with an increase in TMJ pain only up to a certain stage (Va). Then, the joint pain occurs less frequently, but the sounds are more frequent. It was confirmed that, although they occurred in a high percentage in all classes, muscle pain and occlusal interference are not indicators of TMJ damage.

## Data Availability

The data presented in this study are available on request from the corresponding author or from first author representing dental clinic. Data available on request due to restrictions privacy.
